# Principal Component Analysis Reveals the Proximal to Distal Pattern in Vertical Jumping Is Governed by Two Functional Degrees of Freedom

**DOI:** 10.3389/fbioe.2019.00193

**Published:** 2019-08-08

**Authors:** Emily J. Cushion, John Warmenhoven, Jamie S. North, Daniel J. Cleather

**Affiliations:** ^1^Faculty of Sport, Health and Applied Science, St Mary's University, Twickenham, United Kingdom; ^2^Exercise and Sports Science, Faculty of Health Sciences, The University of Sydney, Lidcombe, NSW, Australia

**Keywords:** principal component analysis, vertical jumping, degrees of freedom, constraints, proximal to distal pattern

## Abstract

The successful completion of motor tasks requires effective control of multiple degrees of freedom (DOF), with adaptations occurring as a function of varying performance constraints. In this study we sought to compare the emergent coordination strategies employed in vertical jumping under different task constraints [countermovement jump (CMJ) with arm swing-CMJas and no arm swing-CMJnas]. In order to achieve this, principal component analysis (PCA) was conducted on joint moment waveform data from the hip, knee and ankle. This statistical approach has the advantage of analyzing the whole movement within a time series and reduces multidimensional datasets to lower dimensions for analysis. Both individual and group analyses were conducted. For individual analysis, PCA was conducted on combined hip, knee, and ankle joint moment data for each individual across both CMJnas (thirty-eight participants), and CMJas (twenty-two participants) conditions. PCA was also performed comparing all data from each individual across CMJnas and CMJas conditions. The results revealed a maximum of three principal components (PC) explained over 90% of the variance in the data sets for both conditions and within individual and group analyses. For individual analysis, no more than 2PCs were required for both conditions. For group analysis, CMJas required 3PCs to explain over 90% of the variance within the dataset and CMJnas only required 2PCs. Reconstruction of the original NJM waveforms from the PCA output demonstrates a greater loading of hip and knee joint moments to PC1, with PC2 showing a greater loading to ankle joint moment. The reduction in dimensions of the original data shows the proximal to distal extension pattern in the sagittal plane, typical of vertical jumping tasks, is governed by only 2 functional DOF, at both a group, and individual level, rather than the typically reported 3 mechanical DOF in some forms of jumping.

## Introduction

In both day-to-day and sporting contexts, human beings consistently perform complex motor actions. Whether this is walking up and down flights of stairs at home or performing jumping actions in sport, people are able to perform such skills with relative ease. Yet underpinning these observable actions is a need to coordinate and control multiple degrees of freedom (DOF), referred to as “the degrees of freedom problem” by Bernstein (1967). With so many DOF within the motor system, there are an almost limitless number of coordination patterns available to the performer. However, according to theoretical principles from ecological psychology, and dynamical systems theory, it is proposed that the performer seeks to satisfy the goal of the task whilst operating under continually interacting task, environmental, and organismic constraints, through which coordinated patterns of behavior emerge (Newell, 1986; Hristovski et al., 2006; Orth et al., 2018). Critical, therefore, in successfully completing such tasks is the need to control the “redundant” DOF (Bernstein, 1967; Latash, 2000). These “redundant” DOF, however, provide important functionality as they enable adaptation of emerging actions to the varying performance constraints under which they are operating. For example, Chow et al. (2008) demonstrated that participants adapted their use of limb segments to continue successfully performing a soccer chipping task whilst the task constraints (which we define here as goals or rules which are imposed that influence the movement) were manipulated. Such a finding evidences how the redundant DOF allow for functional variability and for performers to adapt to ever changing performance constraints. Whilst there has been much work within this area it is common for only kinematic changes under varying constraints to be considered (e.g., Ketcham et al., 2002; Seifert et al., 2007; Majed et al., 2017). However, analyzing kinetic factors, such as forces and joint moments, would provide a more complete understanding of how emerging behaviors are shaped as a function of the task constraints under which the skill is performed.

Explaining the control strategies adopted by performers has been a topic within the motor control literature for many years (see Dufek et al., 1995; Bates, 1996; James and Bates, 1997; James et al., 2003, 2014; Nordin and Dufek, 2019). It has been suggested that certain movement features are more important to satisfy the goal of the task, and therefore these features are less likely to show variance between repetitions or from person to person (Scholz and Schoner, 1999; Scholz et al., 2000). This has been demonstrated using several tasks (see Button et al., 2003; Seifert et al., 2011; Nordin et al., 2017). For instance, elbow angle at ball release in a basketball shooting task showed lesser variability between individuals of varying skill levels, compared to other features such as wrist angle at ball release (Button et al., 2003), which indicates that elbow angle at ball release is an important movement feature for successful performance in this task. Identification of such movement features aids in developing our understanding of motor control strategies employed to govern movements under varying task constraints.

Traditional approaches to the study of human movement have typically relied on selecting discrete data points for analysis. However, these methods may not provide a complete representation of the complexities of motor actions due to data being discarded outside of those discrete points. Instead, methods which analyse data across the full temporal cycle of the movement, such as principal component analysis (PCA), may prove more informative (Richter et al., 2014). The attraction of PCA is that large amounts of data across movement cycles can be analyzed whilst reducing the dimensionality of data to features of importance (Daffertshofer et al., 2004). This statistical approach has been used to determine key characteristics of movement tasks (see for example Soechting and Flanders, 1997; Kollias et al., 2001; Lamoth et al., 2006; Dona et al., 2009; Witte et al., 2010; Federolf et al., 2013; Nordin and Dufek, 2016a) as well as determining similarities and differences between groups through data filtering (see for example Deluzio et al., 1997; Deluzio and Astephen, 2007; Laffaye et al., 2007; Lee et al., 2009; Federolf et al., 2013; Verrel et al., 2013). Despite the growth of PCA in sports sciences recently, its application is still in its relative infancy. In particular, fewer researchers have used this approach to determine coordination patterns within movements or to compare movements under differing task constraints (see Forner-Cordero et al., 2005; Lee et al., 2009; Nordin and Dufek, 2016b; Majed et al., 2017 for some exceptions). Therefore, applying a statistical approach such as PCA can be useful in reducing large datasets to key movement features and may be more effective in showing how movement emerges under changing constraints.

Vertical jumping is an interesting motor task to study as it requires the effective coordination of the upper and lower limbs to raise the center of mass vertically, with a clear goal of jumping for height (Raffalt et al., 2016). Vertical jumping also represents a task which can be easily manipulated to provide insight into how emergent behaviors adapt to differing conditions. Analysis of movements under constrained conditions can lead to a better understanding of the key mechanisms for efficient movement, such as the requirement to keep balance in a sit to stand task (Scholz et al., 2001) or the accommodation strategies with increased task demands (Nordin and Dufek, 2016b, 2017). With this in mind, this study used a vertical jumping task and asked participants to complete this action under two different task constraints (with arm swing and without arm swing), with the aim of understanding how motor control strategies, and emergent coordination patterns were affected by manipulation of these constraints. To fully understand these coordination strategies and overcome the limitations associated with traditional analysis methods, we employed PCA to determine the principal components (PCs) that characterize vertical jumping movement patterns both between and within individuals and across the two task constraints. Specifically, we aimed to answer the question of the number of PCs expected to describe either a single jump, repetitions of a jump from an individual, and across the whole group. Typically, lower limb coordination in jumping tasks is described as an extension of the lower limbs in a proximal to distal fashion, with the peak moment progressing temporally from hip, to knee, and then ankle (Bobbert and van Ingen Schenau, 1988). A moment curve is typically bell-shaped and unimodal (examples can be seen in **Figures 6**, **7**). If the timing of peak moments and the shape of the moment curves are independent of one another it would be expected that 3 PCs would be required to represent the 3 moment curves of a particular jump (that is 3 functional DOF). If the peaks of the 3 moment curves occur at different time points along the curve, then there needs to be more than one PC score.

## Materials and Methods

### Participants

Thirty-eight healthy individuals (males = 24, females = 14) volunteered to take part in this study (mean ± SD; age = 27 ± 5 years, height = 174.4 ± 9.0 cm, body mass 78.1 ± 14.1 kg). Participants were free from musculoskeletal injuries and were provided with details of the study before written informed consent was obtained. The experimental procedure was approved by the ethics sub-committee at the institution where the research took place.

### Procedure

Participants were required to attend one data collection session. This involved the collection of anthropometric measures (height and weight), before each participant was provided with a standardized shoe according to their shoe size. Eighteen reflective markers were placed on the pelvis and on the right lower limb. Data from the right limb was used for further analysis in accordance with previous work from Cleather et al. (2013). Markers were placed on the right and left anterior superior iliac spine and posterior superior iliac spine, lateral and medial femoral epicondyle, apex of lateral and medial malleolus, posterior aspect of calcaneus, tuberosity of fifth metatarsal, and head of second metatarsal. Three additional markers placed on rigid plates were attached to the mid-thigh and anterior tibial shaft, with an additional marker attached to the top of the foot. Kinematic data was collected using a Vicon motion capture system (Vicon MX System, Nexus 2.2 software, Vicon Motion Systems Ltd, Oxford, UK) with 14 LED cameras tracking the reflective markers at a sampling frequency of 200 Hz. Kinetic data was collected via two force plates positioned flush to the laboratory floor (Kistler Type 9287BA, Bioware 3.24 software, Kistler Instruments Ltd, Hampshire, UK), at a rate of 1,000 Hz and synchronized with the Vicon system.

Participants completed a standardized warm-up (bodyweight squats, lunges, inchworms, hip rotations, and vertical jumps) prior to completing any vertical jumps. Thirty-eight participants performed a vertical jump task (countermovement jump with no arm swing—CMJnas). For this task, participants were instructed to keep hands in contact with the hip throughout the jump, and to land with one foot on each force plate. Participants were required to complete five maximal effort trials with a self-selected recovery period between each jump to reduce any effects of fatigue. Twenty-two of the participants carried out an additional jumping task. For this task, participants performed a vertical jump with the use of an arm swing (CMJas). Participants were instructed to use an arm swing to aid in jumping performance and were required to land with one foot on each force plate. For these participants the order of completion of the two tasks was counterbalanced to prevent order effects. Participants were given a 2 min recovery period between the two tasks.

### Data Analysis

All data was filtered using a 5th order Woltring filter with a cut off frequency of 10 Hz. The propulsive phase of the vertical jump was used for analysis and was defined as being from the point where the right anterior superior iliac spine marker moved below stationary height until take-off (which was defined as the point where the ground reaction force fell to zero). Net joint moments (NJM) in the sagittal plane were calculated for hip, knee, and ankle during this phase using a standard inverse dynamics calculation (Winter, 2005) within the FreeBody software (Cleather and Bull, 2015). Each jump trial therefore had the 3 mechanical DOF described by the NJM.

As trial length varied across each participant, data was spline interpolated and time normalized from 0 to 101 data points. Maximal jump height was analyzed through calculation of the change in displacement of the right anterior superior iliac spine when standing, to the maximum height achieved during the jump (Chiu and Salen, 2010).

### PCA Calculation

PCA was used in this study to extract common patterns of moment production during the vertical jump under two task constraints. Using this approach has the advantage of retaining the spatiotemporal pattern in the time series data whilst detecting coordination patterns both within and between individuals. The fundamental purpose of a PCA is to find a linear transformation that maps the raw data described in its original coordinate frame to a new coordinate frame with orthonormal bases. In the context of data analysis, the coordinate frame for the raw data is defined by the measured variables, but these variables may have some degree of correlation with one another. The new coordinate frame that is given by the PCA will be defined by a set of new uncorrelated variables called the principle components (PC). For instance, for a dataset consisting of *p* variables observed at *n* different time-points the raw data can be described by the *n* × *p* matrix *X* where the columns of *X* are the individual variables, and the rows represent each observation (time-point). The transformation *U* then maps the raw data to the new coordinate frame defined by the PCs, such that the raw data in the new coordinate frame *Z*, is given by *Z* = *XU*.

In biomechanics, PCA has sometimes been used to compare time-normalized waveforms. For instance, a waveform can be described by the value of a variable sampled at 1% intervals over the course of a movement. Typically, we have multiple waveforms (cases) for one particular biomechanical variable which we want to compare—for instance, if we want to compare the curves of different individuals. In this instance, as outlined extensively in Deluzio and Astephen (2007), the individual time-points have often been considered as variables and cases (observations) considered to be the individual curves being analyzed. Thus if we had *p* different curves for a given variable, that each consists of 101 time-points, we would perform the PCA on the *p* × 101 raw data matrix *X*^*T*^. In the present study we have adopted a more intuitive approach to study waveforms that is closer to the traditional description of PCA. We have treated the time normalized intervals as observations, and the individual curves (cases) as variables. Thus, for our *p* different curves for a given biomechanical variable sampled at 101 time-points we perform the PCA on the 101 × *p* raw data matrix *X*. This approach has been used previously (see Borzelli et al., 1999).

It should be noted that in the method of Deluzio and Astephen (2007), a separate PCA is performed for each of the measured variables. In contrast, in this study we have included all of the measured variables within the same PCA. For instance, in this study, for an individual subject we have time-series for 3 biomechanical variables (hip, knee, and ankle NJM) measured in 5 different jumps. Using the method of Deluzio and Astephen (2007) we would perform 3 PCAs on the 3 5 × 101 matrices representing each joint. Instead, in this study we perform only one PCA on the 101 × 15 matrix which includes all of the individual's data.

One common application of PCA is to reduce the dimensionality of the data being analyzed—that is to reduce the number of variables under consideration to a smaller set which describe the majority of the variance in the data. The advantage of the method we have adopted here is that the coordinate frame in which the data is described is defined by all of the cases for all of the measured joints (NJM). This means that the PCA produces a reduced new coordinate frame that can be used to describe all cases and joints. This therefore means that the reduced coordinate system represents the number of functional degrees of freedom that are present in the movement being analyzed. Conventional approaches to using PCA would require each variable (e.g., hip NJM, knee NJM, ankle NJM) to be analyzed separately. This would limit the ability to effectively interpret the functional DOF describing the movements. Thus, for the present study PCA was used in this manner to extract common patterns of moment production during the vertical jump under two task constraints. Only those PCs that cumulatively explained over 90% of the variance in the data set were retained and used in further analysis (Jolliffe, 2002).

Prior to running the PCA, all data was normalized to the peak hip joint moment of each trial (Jolliffe and Cadima, 2016). Analysis of the data was performed on both an individual (termed analysis 1), and group (termed analysis 2) basis (as has previously been conducted within the literature, e.g., Nordin and Dufek, 2016a,b, 2017) and the PCs from the individual (PCi), and group (PCg) analyses are indicated by subscripts as shown. For the individual analysis a PCA was carried out for each individual separately, which contained hip, knee and ankle NJM data combined (101 × 15 matrix for each individual containing 15 columns representing 5 hip, 5 knee, and 5 ankle joint moment time series with 101 data points). This was performed for both CMJnas and CMJas conditions. For the group analysis, all NJM data from each participant were combined and subjected to a PCA (CMJnas: 101 data points × 567 joint moment time series, CMJas: 101 data points × 330 joint moment trials). A further PCA was performed to compare CMJnas and CMJas data with only the participants who had performed both jumps (101 data points × 672 joint moment time-series). This is referred to as analysis 3. Finally, an additional group level PCA was conducted using only the first 2 PCs obtained from the individual PCA (CMJnas: 101 data points × 76 PCs, CMJas: 101 data points × 44 PCs; hereafter called analysis 4).

The output of each PCA was a matrix (henceforth named the coefficients matrix) where each column gives the coefficients of a PC, and the PCs are ordered in terms of the amount of variance they explain. The coefficients matrix thus gives the transformation of the raw data into the variable space defined by the PCs. Thus, the matrix obtained by multiplying the raw data matrix by the coefficients matrix is a matrix describing the time-series of the values for each PC. These time series are reported as the PC scores in this study. Similarly, the raw data can be reconstructed based upon a limited number of PCs by transforming the selected number of PCs back to the variable space defined by the raw data. This was performed for CMJnas data as an example. All PCA calculations were performed in Matlab (The MathWorks, Inc., MA, version 2017a).

### Statistical Analysis

Further statistical analysis was performed to compare the variance explained by each PC attained from the individual analysis of both CMJnas and CMJas. Group differences between PC1, PC2, and the sum of PC1 and PC2 were assessed using an independent sample *t*-test. The alpha level was set at *p* < 0.05.

## Results

**Figure 1:**
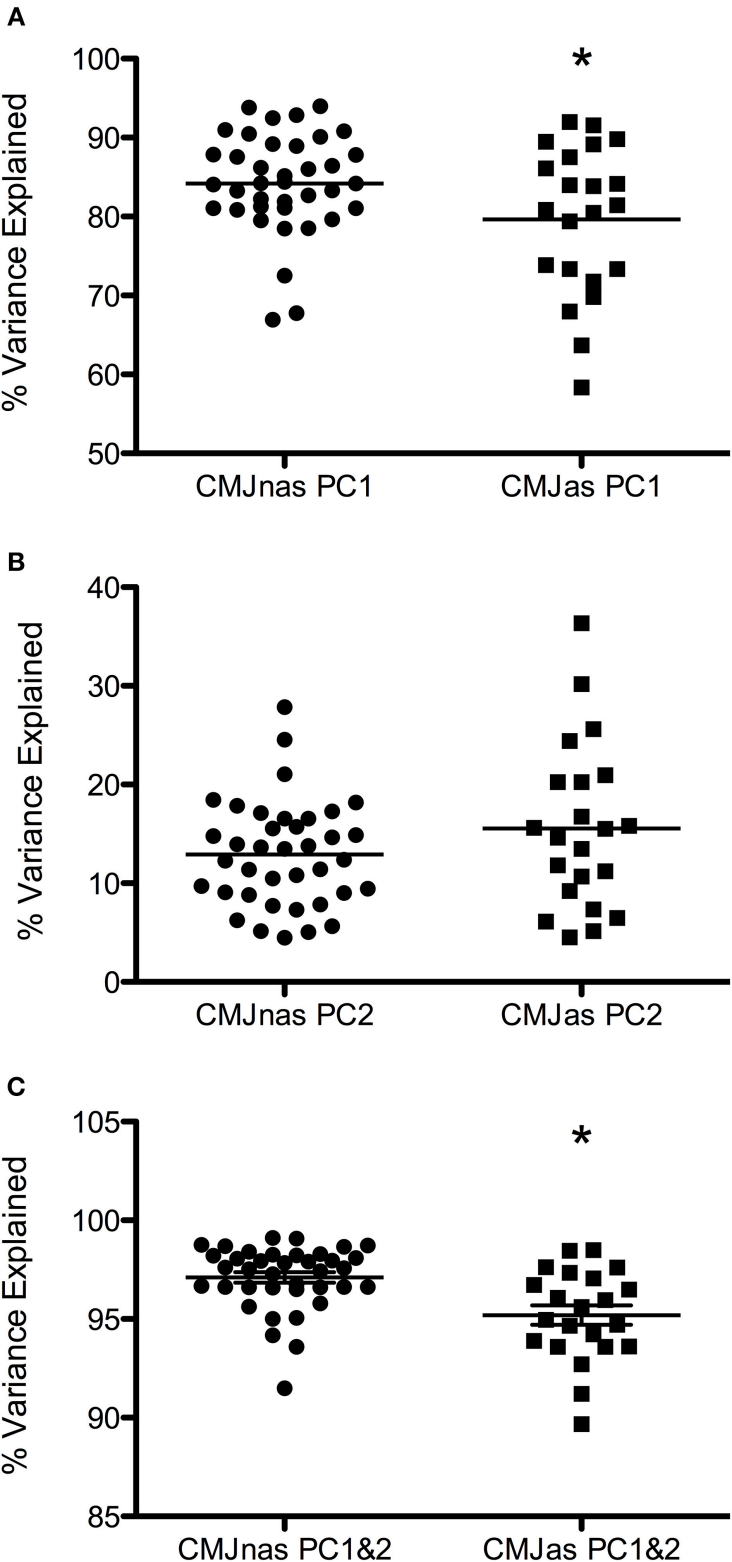
Variance explained by **(A)** PC1i, **(B)** PC2i, and **(C)** the sum of PC1i&2i for CMJnas, and CMJas. Horizontal line represents group mean. i = individual PCA analysis. *Indicates significant difference from CMJnas.

**Figure 2:**
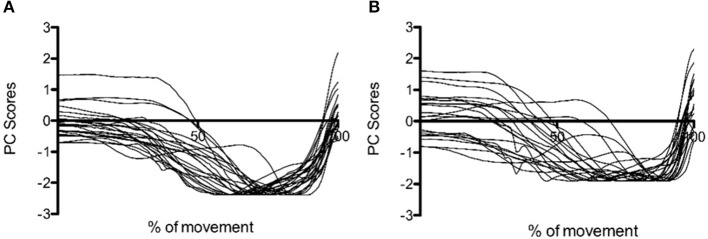
PC1i scores for **(A)** CMJnas and **(B)** CMJas from 22 participants completing both conditions. Data is normalized to peak value. i = individual PCA analysis.

When considered individually, only one or two PCs were required to describe at least 90% of the variance in the data. Data from each individual PCA showed PC1i and the sum of PC1i, and 2i explained a significantly greater amount of the variance in CMJnas than CMJas (*p* = 0.05, *p* = 0.00, respectively; Figure 1). The greater variation in PC1i scores for CMJas can be seen in Figure 2.

**Figure 3:**
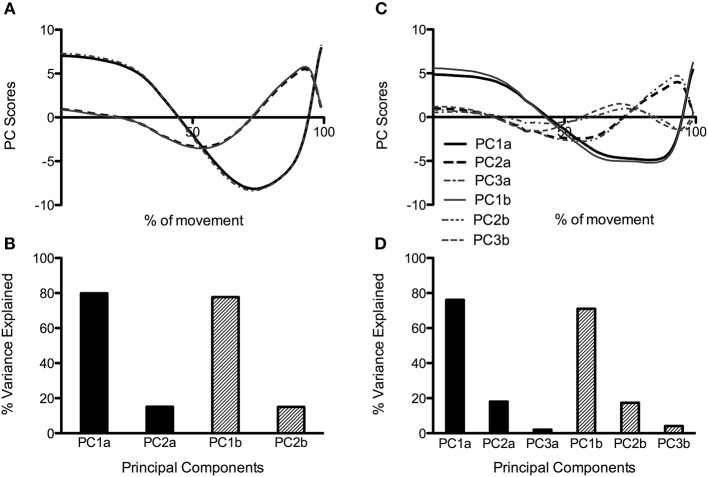
PC waveforms along with percentage of explained variance for CMJnas **(A,B)** and CMJas **(C,D)** conditions. Data in **(C)** has been flipped to a negative peak to more clearly show comparison between the shape of waveforms in **(A,C)**. PC1, 2, and 3a represent data obtained from PCA using only the first two PCs from each individual (analysis 4). PC1, 2, and 3b represent data obtained from group PCA for CMJnas, and CMJas (analysis 2).

When all joint moment data was combined in the same PCA, three PCs were retained for the CMJas condition, and two were retained for the CMJnas condition (Figure 3). Figure 3 also illustrates waveforms from the group analysis PCA that just retained the first two PCs for each individual (analysis 4).

**Figure 4:**
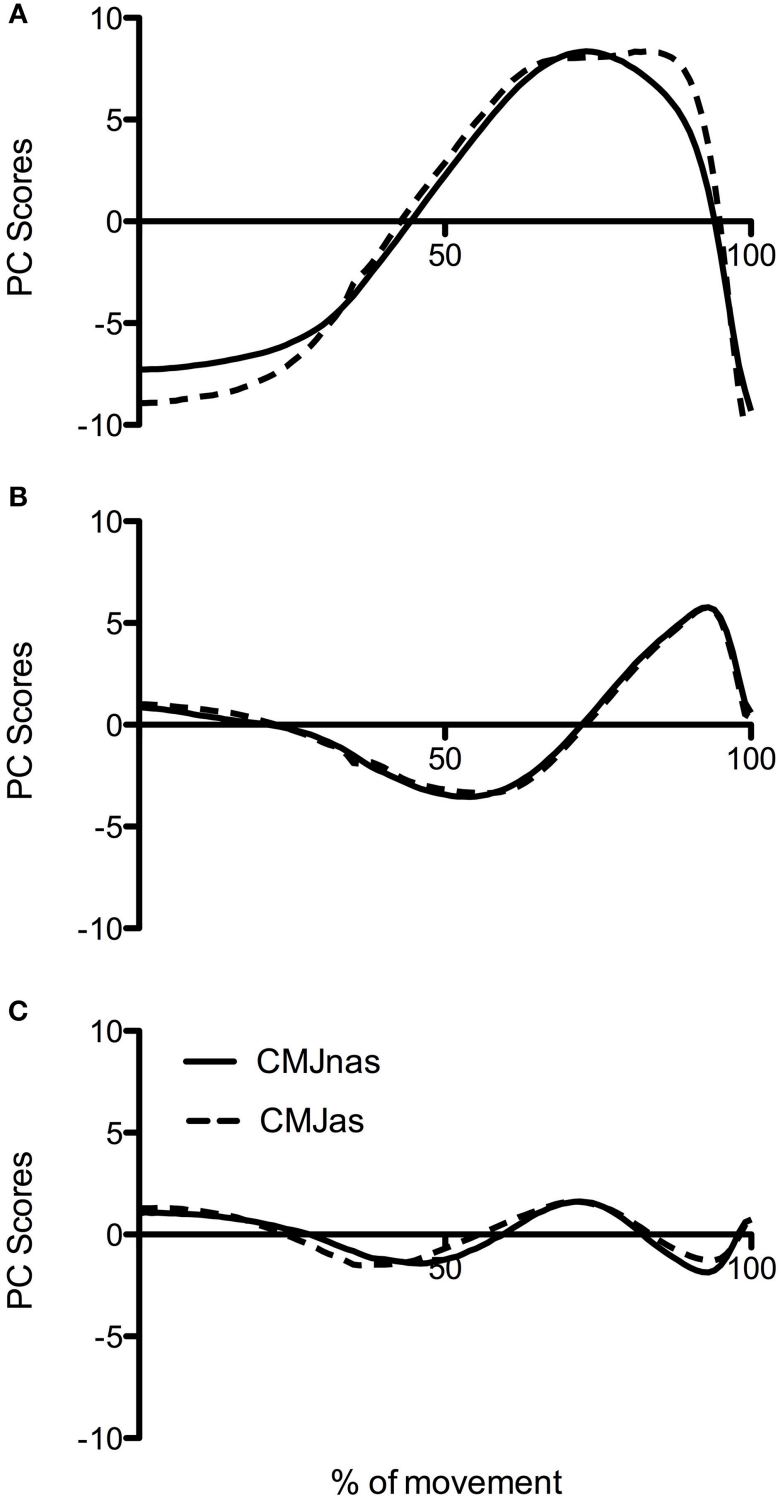
Comparison of PC waveforms for CMJnas and CMJas for the 22 participants who completed both jumps: **(A)** PC1, **(B)** PC2, and **(C)** PC3. CMJas waveforms have been flipped and peaks matched to show differences in temporal pattern.

To make a robust comparison between jumps at the group level, data was reanalyzed with only those participants who completed both CMJnas and CMJas conditions (Figure 4, analysis 3). This highlighted the same trend with CMJas requiring three PCs and CMJnas requiring only two PCs to describe over 90% of the variance. Waveforms for each jump condition were similar to those observed in Figure 3.

**Figure 5:**
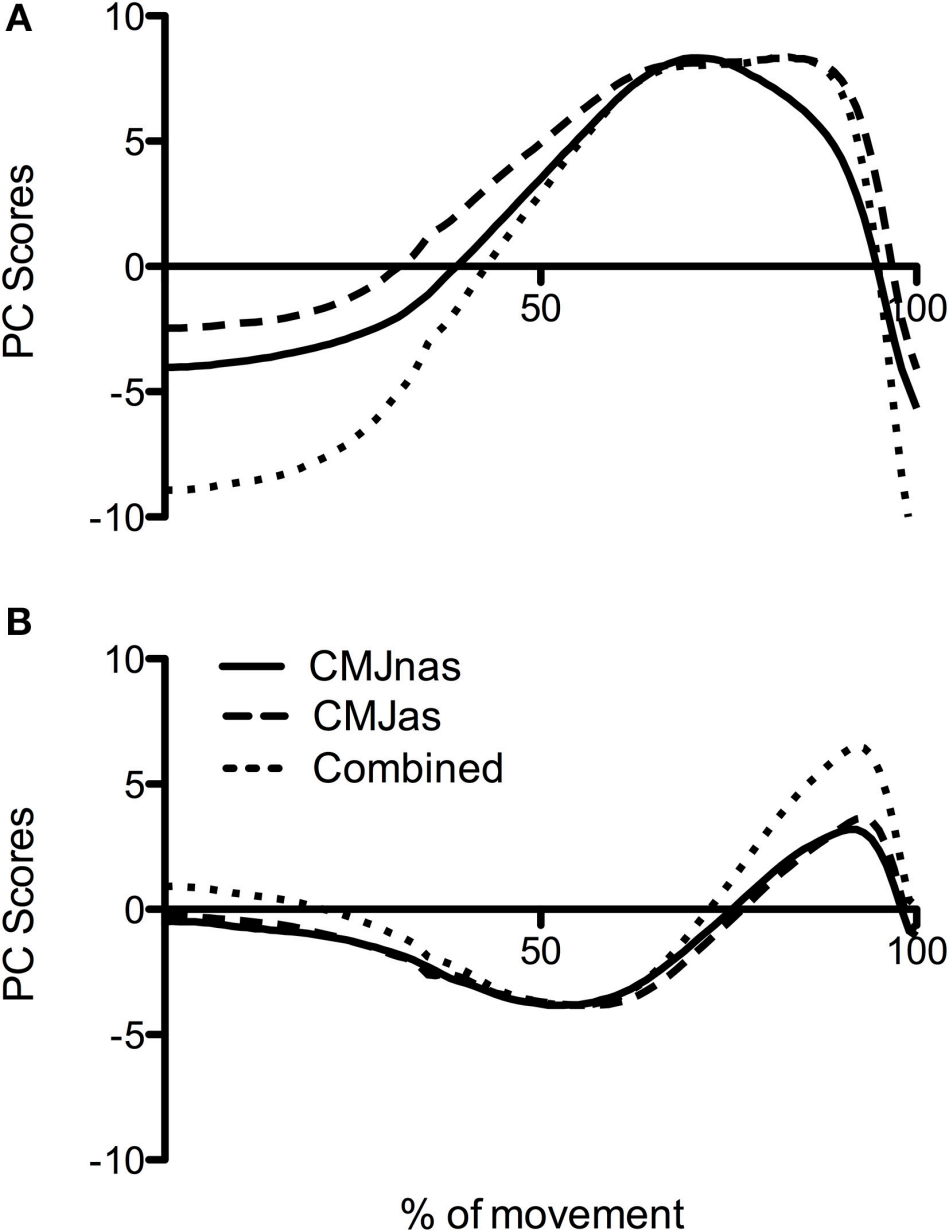
Comparison of PC waveforms for CMJnas and CMJas for the 22 participants who completed both jumps along with PC waveform of combined CMJnas and CMJas analysis (analysis 3): **(A)** PC1, **(B)** PC2. CMJas waveforms have been flipped and data peaks matched to show differences in temporal pattern.

An analysis was also conducted with only those participants who completed both CMJnas and CMJas conditions with all joint moment data combined (Figure 5). Only two PCs were required to explain over 90% of the variance.

**Figure 6:**
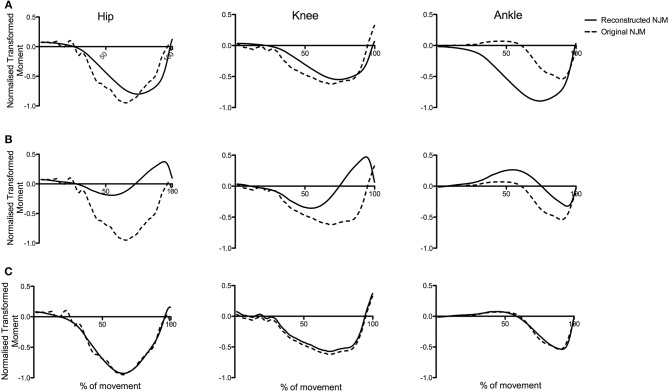
Original NJMs (raw data) for a typical CMJnas compared with reconstructed data using only the first two principal components. The reconstructed curve in **(C)** is the linear sum of the curves in **(A,B)**.

Figure 6 presents an illustrative example of the reconstruction of a typical jump from PC1g and PC2g—and shows how the sum of the time series for PC1g and PC2g describe such a high proportion of the variance in the raw data.

**Figure 7:**
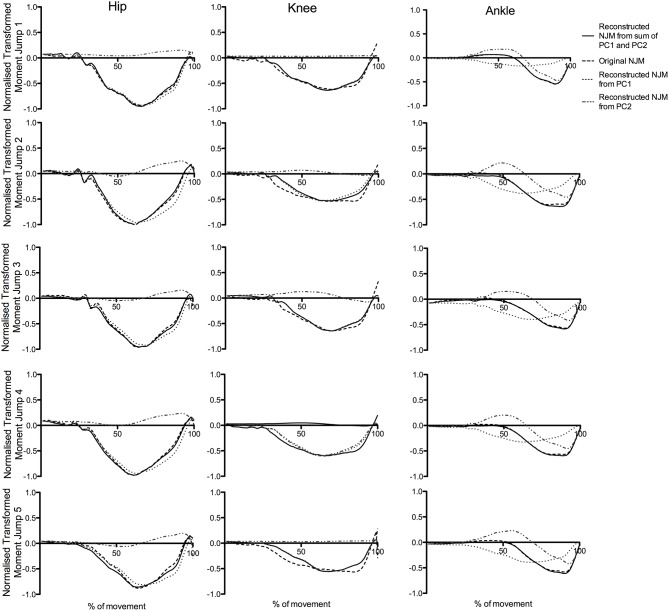
Original NJMs (raw data) for a typical CMJnas compared with reconstructed data using only the first two principal components, for a representative participant across five jump trials.

Figure 7 presents an illustrative example of the reconstruction of a series of five jumps from a representative participant performing CMJnas. The figure shows the sum of the time series for PC1i and PC2i describe a high proportion of the variance in the raw data.

## Discussion

The purpose of this study was to use PCA to characterize movement strategies, through reducing a large data set of hip, knee, and ankle NJMs measured during vertical jumping. The main results of this study were that for all comparisons considered here, 90% of the variance in the raw data could be described by at most 3 PCs, and in many cases by 2. Whilst there are multiple sources of variation within the dataset (e.g., joint, trial, individual), we suggest that the PCs here largely describe the variance in joint moments during jumping, and suggest that the joint moment production both within, and between individuals and within each task, share similar structural patterns. Further to this, the dimensionality of sagittal plane lower limb extension in some forms of vertical jumping can be reduced to only 2 functional DOF. This is an important result—it indicates that simply providing the participants with the task constraint “jump as high as you can” produced a markedly similar pattern, and that between and within participant variation amounted to <10%. This is a highly non-trivial result and provides insight to the nature of motor control within this task—although it has previously been suggested that during jumping, people exhibit a characteristic proximal to distal pattern of joint moment production (Bobbert and van Ingen Schenau, 1988), the degree of similarity has not been emphasized.

When considering the group and individual analysis for CMJnas only 2 PCs are required to explain over 90% of the variance (Figure 3). This would suggest the relationships between curves are not just unique to specific individuals but are demonstrated by the whole group. If this was not the case further PCs would be required to describe the movement. For CMJas an additional PC was required at the group level in order to capture 90% of the variance in the data (see Figure 3), although it was not required at the individual level. These results are consistent with previous research findings which have also looked to determine how the high dimensionality of human movement can be reduced to low dimensional states (see Soechting and Flanders, 1997; Borzelli et al., 1999; Balasubramaniam and Turvey, 2004; Witte et al., 2009; Nordin and Dufek, 2016a; Majed et al., 2017), For example, (Santello et al., 1998) have shown that in a hand grasping task only two PCs were required to describe over 80% of the variance in the data, demonstrating few synergies govern the production of hand motion in the specific grasping task. Equally, in a sit to stand task only two PCs were required to describe the task from six components of ground reaction force data (see Borzelli et al., 1999).

It is interesting to note that at only the group level an additional PC was required to describe over 90% of the variance within the dataset for CMJas. The need for the third PC is indicative of the greater variability in the CMJas data. This greater variability is seen both within individuals (see Figure 1) and between individuals (see Figure 2), where a significant difference in explained variance for PC1 was observed between CMJnas and CMJas. In contrast, in analysis 4 (which essentially eliminates the effect of within individual variation on the group scores) only 2 PCs are required to explain 90% of the variance. This improvement in predictive value is gained in the variance explained by PC1g. It is interesting to consider the shape of the PC3g score and think about the linear sum of the PC1g score with PC3g score. In particular, the effect of adding PC3g to PC1g is to make the curve less flat and to make it more peaked toward the end of the jump. This type of variation is also seen between individuals (Figure 2). It is also worth noting that the requirement for a third PC for the group analysis provides further support for the fact that the reduction to 2 PCs seen for CMJnas is not trivial. The increase in variability within the CMJas data could be related to unfamiliarity with the task or complexity of the task for the participants involved. It has previously been demonstrated an increase in complexity of a task results in an increase in dimensionality and a reduction in explained variance within the first principal component (Zago et al., 2017). Whilst participants within this study were familiar with jumping, the requirement to use an arm swing may have added complexity to the task such that they were required to explore movement options to a greater extent than in the constrained CMJnas condition.

Results from the present study suggest the first PC represents the hip and knee joint moment whereas the second PC represents the ankle. Reconstruction of the raw joint moments from the PCA (Wootten et al., 1990; Troje, 2002) can aid in visualizing this. The reconstruction of the moment curves for a representative individual (see Figure 6), shows the score for PC2i featuring heavily in the linear sum of the reconstructed curve for the ankle moments. Similarly, the first PC curve shares a similar pattern to either the hip or knee joint moment curve—this varied depending on the individual (for the individual in Figure 6 it was hip-like). The relationship between the two PCs can also be seen in Figure 7. Consider, for instance, the ankle moments and compare jump1 to jump4. Here jump4 shows a flatter peak compared to jump1 and a much wider curve. Consideration of the 2 PC scores shows how these 2 quite different ankle moment curves can still be constructed. In particular, the scores for PC1i, and PC2i are out of phase, however the difference in timing between them is such that they can still be summed to produce all the different ankle curve combinations. To put this another way, given the differences in the shape of the ankle curves we know that at least 2 PCs would be needed to reconstruct them. However, the 2 PCs that we obtain not only allow all of the ankle moment curves to be created, but also all the hip and knee moment curves. This synergy implies a relationship between the different joint moment curves that persists across jumps.

This concept of synergy is not unique to this study and demonstrates an approach of the system to optimize control of motor tasks (see Santello et al., 1998; Jaric and Latash, 1999; St-Onge et al., 2004; Todorov and Ghahramani, 2004; Latash et al., 2007; Latash, 2010; Kipp et al., 2012; Nordin and Dufek, 2017). This organization of movement to synergies may occur due to mechanical constraints (defined as limits in the options of motion that can be achieved due to the mechanical configuration of the human body) on the system that determine the movement pattern—so called mechanical intelligence (Blickhan et al., 2006). Within this study it is suggested that the proximal to distal pattern of moment production (in the sagittal plane) during vertical jumping might be governed by only 2 functional DOF and this may be related to mechanical constraints. For instance, Cleather (2018) has recently shown how the geometry of the patellofemoral joint alone can explain over 90% of the variance in the relative timing of femoral and tibial segment moments (Cleather, 2018). To put that observation in terms of the findings reported here, the patella could provide a mechanical constraint that reduces 2 functional DOF to 1—the hip and knee moments are largely dependent on one another. This would then represent a potential explanation for the remarkably consistent timing of moment production here. It seems more credible that such consistency would be produced by a mechanical feature of human anatomy that is shared by all (a shared organismic mechanical constraint), than it being the result of everyone learning a pattern of motor coordination during their development that is so markedly consistent. A similar conclusion of mechanical constraints reducing the dimensionality of movement patterns has been proposed in a similar, albeit much slower task of sit to stand (Borzelli et al., 1999) as well as in drop landings (Nordin and Dufek, 2017).

An additional comparison between CMJnas and CMJas was made between only those individuals who had completed both forms of the jump (Figures 4, 5, analysis 3). This comparison demonstrates that the PC2g scores are effectively the same. This can be interpreted as suggesting that the ankle is being used in the same way, and that the potential effect of the ankle on the relative timing of hip and knee NJM is also the same. However, there is a notable difference in the PC1g scores. Specifically, Figure 4A shows PC1g curves between each jump. This shows a PC1g curve for CMJas to exhibit a flatter peak along with a greater area under the curve compared to the CMJnas condition. This is likely to be reflective of the use of the arm swing to slow joint extension and thus provide greater time for the musculature to develop force (Feltner et al., 2004; Domire and Challis, 2010). Previous literature (e.g., Lees et al., 2004; Hara et al., 2006) has suggested that the use of the lower limb in CMJnas and CMJas is distinct. The results reported here suggest that although this is true, the motor control strategy only differs in terms of the first PC, and that there is still a striking similarity between the movements.

Whilst interesting and novel findings have been identified within this study, limitations of this work should be highlighted. Although statistically rigorous procedures have been employed, the analysis of the results is in some parts interpretative. This is a limitation of trying to describe the mechanics of movement from statistical techniques—although it should be noted that we are not alone in using this technique to tease out different sources of variation (e.g., Daffertshofer et al., 2004). In particular, there are multiple sources of variation—joints (hip, knee or ankle), participant, trial, and type of jump (with or without arms). Our contention in this paper is that the PCs primarily represent the variation in moment production between different joints. This can be inferred based upon the following reasoning. Firstly, the PCA analysis was first performed considering each individual separately—thus eliminating inter-individual variability—this analysis produced similar PC1i and PC2i for each participant suggesting that the PC1g and PC2g are not produced by inter-individual variability. Secondly, the group PCA analysis was performed on both the whole data set, and on a reduced data set comprising the resulting PCs from the individual analysis. Inter-trial variation for each individual would be to a large part mitigated in the latter data set, yet there was little difference in the results of these two analyses (Figure 3). This suggests that inter-trial variability is not a major factor in the group PCs. Thirdly, the variation based upon type of jump was quantified by the comparison of CMJas and CMJnas in Figure 4, and the same PCs were found for both jump types. Having eliminated 3 of the 4 sources of variation, we suggest that the PCs from the group analysis describe variation in joint moment production. Of course, this contention has not been “proven” statistically, but, we argue, is the only reasonable explanation. Furthermore, Figures 6, 7 demonstrate the reconstruction of joint moments from the PCA. This shows the similarity of the reconstructed curves to the original raw joint moment curves. If variance was predominantly from individual differences in jumping style, the ability to so closely reconstruct the moment curves would likely not occur. In addition, whilst the focus of this research was on the analysis of the lower limb during jumping, based on their large contribution to the movement, future research would benefit from conducting a whole-body analysis to determine the dimensionality of the system.

In conclusion, the current study has shown the applicability of using a PCA to analyse complex multi-joint tasks and successfully compare between similar movements. As discussed, there are several key outcomes from the use of a PCA within this study. This analysis method was effective in reducing the statistical and mechanical dimensionality of the data in jumping tasks and showed a maximum of three PCs were required to describe 90% of the variance in the original data set for a group analysis. Specifically, 3 PCs were required to explain the variance within CMJas group data and only 2 PCs were required for CMJnas group data. The first PC has a greater loading for hip and knee joint moments, whereas the second PC has a greater loading for the ankle joint moment. The results suggest the proximal to distal extension pattern in the sagittal plane typical of vertical jumping tasks is governed by only 2 functional DOF rather than the previously reported 3 mechanical DOF. This study adds to the motor control literature suggesting that despite the redundancy within the system coordinated movements are produced based on task and mechanical constraints. Importantly, the results suggest vertical jumping is controlled by 2 functional DOF at both an individual and group level. We suggest the reduction in dimensionality of the movement may be mechanically driven by human anatomy.

## Ethics Statement

All subjects gave written informed consent in accordance with the Declaration of Helsinki. The protocol was approved by the ethics sub-committee of St Mary's University.

## Author Contributions

EC, JN, and DC were involved in the design of the study. EC carried out all data collection. All authors were involved in the interpretation and final write up of the study.

### Conflict of Interest Statement

The authors declare that the research was conducted in the absence of any commercial or financial relationships that could be construed as a potential conflict of interest.
